# Comparing the kinematics related to inflicted head injury between violent shaking of a 6-week-old and a 1-year-old infant surrogate

**DOI:** 10.1098/rsos.251251

**Published:** 2025-11-26

**Authors:** Kim Hutchinson, Arne Stray-Pedersen, Jenny Dankelman, Ajay Seth, Arjo J. Loeve

**Affiliations:** ^1^ Department of BioMechanical Engineering, Delft University of Technology, Delft, Zuid-Holland, The Netherlands; ^2^ Department of Forensic Sciences, Oslo University Hospital, Oslo, Oslo, Norway; ^3^ Department of Forensic Medicine, University of Oslo, Oslo, Norway; ^4^ Co van Ledden Hulsebosch Centre for Forensic Science and Medicine, Amsterdam, Noord-Holland, The Netherlands

**Keywords:** biomechanical model, inflicted head injury by shaking trauma, head kinematics, abusive head trauma, child abuse

## Abstract

Annually, 14–41 per 100 000 infants get mildly to lethally injured or severely disabled through violent shaking. The incidence and mortality of inflicted head injury by shaking trauma (IHI-ST) are highest in the early months and decrease with age. This may partly be due to the age-related physical characteristics of infants. Younger, smaller infants are more vulnerable owing to their size and material properties. In addition, from basic biomechanics, it is expected that larger or heavier infants may be more difficult to fiercely shake and will exhibit different motion patterns when being shaken violently. Therefore, the aim of this study was to compare the kinematics of shaking a smaller versus a larger infant dummy. We recorded the kinematics of two dummies, representing a 6-week-old and a 1-year-old, while they were violently shaken by volunteers. We found that participants induced higher head and torso accelerations when shaking the 6-week-old, than with the 1-year-old dummy. Moreover, higher peak sagittal angular accelerations coincide with smaller radii of rotation in the 6-week-old than in the 1-year-old. Because it has been suggested in the literature that sagittal angular acceleration of the head is an important mechanism in inducing the injuries associated with IHI-ST; the results of this study show that shaking a smaller/younger infant is more likely to cause the kinematics possibly responsible for IHI-ST.

## 1. Introduction

Inflicted head injury, commonly known as abusive head trauma, is the leading cause of serious and fatal head injury in infants [1–3]. In infants these injuries are most commonly caused by blunt force trauma, inflicted head injury shaking trauma (IHI-ST), or a combination of both [4,5]. IHI-ST is defined as head injury resulting from rapid repetitive acceleration/deceleration of a victim’s head, usually induced through forceful back-and-forth movement of the victim’s torso in its sagittal plane. It has been estimated that IHI-ST is diagnosed annually in 14–41 per 100 000 young infants, with the highest incidence in the first few months and decreasing with age [6–8]. Peaks in mortality resulting from inflicted head injury have been found in the first 1–4 months of age [1,9,10]. IHI-ST is associated with injuries including encephalopathy, subdural haemorrhages and retinal haemorrhages [5,11]. However, no set of injuries is considered uniquely characteristic for IHI-ST, since these injuries can each have other causes. Therefore, the diagnosis of IHI-ST also relies on the exclusion of other causes, the victim’s (clinical) history, and witness or perpetrator statements. Furthermore, the exact injury mechanisms of IHI-ST remain unknown. As a result, court cases pertaining to potential victims of IHI-ST are often inconclusive, with potentially severe adverse consequences such as falsely convicting innocent caregivers or leaving a vulnerable infant in the care of abusive ones.

Owing to obvious ethical limitations of using infants to experimentally study IHI-ST, several modelling approaches have been used instead, including animal, computational and physical models [12–14]. Studies using physical dummy experiments and rigid-body computational models to study shaking usually report gross body kinematic parameters such as linear acceleration and velocity of head vertex and centre of mass (CoM), head angular acceleration and velocity [12,13] or frequency analyses of these kinematics [15]. These studies are often used in an attempt to relate differences in kinematics in various scenarios to differences resulting in injury patterns, for example, in comparison of violent shaking to impact from falls. The measured kinematics are often also the input to computational models; usually, linear motions in the sagittal plane are prescribed on the torso [16–19] or sagittal rotational motions are prescribed directly on the head, with a fixed centre of rotation somewhere in the neck [20–28]. At any instant, a planar motion (be it pure rotation, pure translation or a combination of both) of a rigid body can be described as a pure rotation around a point in space known as the instantaneous centre of rotation (ICOR). In their study on the kinematics of shaking a physical infant dummy, Schiks *et al.* [29] found that the location of the ICOR during shaking is not fixed in the neck, but travels through space over time instead, and that assuming it *is* fixed results in incorrect estimates of measured head kinematics. We refer to the distance from the object’s CoM to its ICOR as its instantaneous radius of rotation (IROR). Thus, a smaller IROR of the head during shaking indicates more rotational motion, while a larger IROR is seen as more translational motion (e.g. rotating around a point close to, versus far away from, the CoM of the head). As such, the IROR is indicative of the nature of the relative motion between the skull and its contents, and therefore of loads on, and deformations of, the brain.

To avoid confusion between the similar appearing abbreviations ICOR and IROR, we define them as follows:

— **ICOR** is the *point* in space around which an object’s motion can instantaneously be described as a pure rotation.
— **IROR** is the *distance* between that point and the CoM of the object.

It has been hypothesized and supported by simulation studies that sagittal angular acceleration may be an important mechanism responsible for torn bridging veins resulting in subdural haemorrhage [11,30–32]. Song *et al.* [28] investigated the effect of linear versus rotational motion of a finite element model of the paediatric eye on the tension stresses in the retina. They found that angular motion, rather than linear, generated much higher tension stress in the retinal layers. The layer of cerebrospinal fluid occupying the subarachnoid space protects the brain during motions that result in high translational acceleration, e.g. impact, through damping. This damping limits both the strain of brain tissue that would result from undamped impact with the skull, as well as the relative motion of the brain with respect to the skull and corresponding strain of bridging veins in case of linear accelerations. However, during angular head accelerations and in particular with small rotation radii, that fluid layer hardly exerts moment on the brain that would prevent its rotation and therefore lubricates the motion of the brain with respect to the skull, which would result in higher strain on bridging veins. A motion described by a very large IROR would result in tissue dynamics similar to those occurring during translation, while a motion with the same angular acceleration but a small IROR would result in the dynamics resulting from rotational motion as described above. Consequently, even head motions that are seemingly less fierce (e.g. lower accelerations) but induce more relative rotation of the brain with respect to the skull (e.g. small IROR) may still be more damaging than apparently fiercer (e.g. higher acceleration) head motions that are mainly translational (e.g. large IROR) [25,30–33]. Therefore, the studies mentioned above suggest that the same angular head acceleration with a small IROR is more likely to result in the types of injuries typically associated with IHI-ST than with a large IROR.

Little is known about the relationship between an infant’s build and the risk of incurring injury as a result of shaking, but several physical factors have been proposed. First, younger infants have a relatively larger head relative to their body size and relatively weaker neck musculature [34]. Jones *et al.* used a rigid-body model of an infant to investigate the effect of neck stiffness on the accelerations of the head during simulated IHI-ST and indeed found that these accelerations increase with decreasing neck stiffness [19]. Second, basic biomechanics predicts that a larger, heavier infant would be more difficult and physically challenging to accelerate fiercely than a smaller, lighter infant. The combination of these factors may result in the motion of the head during shaking being more rotational in nature in younger/smaller infants and more translational in larger/older infants. So, in addition to the psychological explanations that have been posed for the higher incidence of IHI-ST in the first few months of life, the above biomechanical factors could explain a higher incidence of serious injury in younger infants, even if shaking were to occur at the same rate.

The goal of the current study was to compare the kinematics of two infant dummies, representing a younger and older infant, based on the physical characteristics of the dummy and the person shaking. This was achieved by recording the kinematics, including the spatiotemporal variation of the sagittal ICOR for its relevance to shaking, of the head and torso during violent shaking of two infant dummies representing a 6-week-old and 1-year-old infant.

## 2. Methods

### 2.1. Experiment protocol and study population

**Figure 1.**
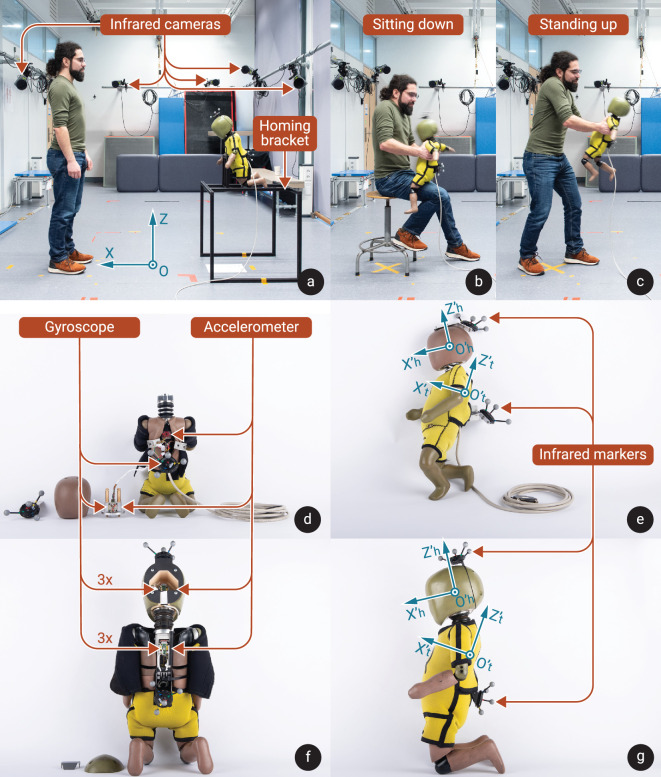
Overview of the experimental set-up and dummies used. The motion capture lab with infrared cameras used for tracking the dummy position, homing bracket that the dummy is placed in at the start and end of each trial, and global reference frame is shown (a). Two postures participants shook the dummy in were sitting down (b) and standing up (c). Accelerometers and gyroscopes integrated into Q0 (d) and Q1 (f) are indicated. The infrared markers used for tracking Q0 (e) and Q1 (g) are indicated, as well as their respective local head and torso reference frames.

Two infant dummy shaking experiments were conducted. The dummies used were the Q-series Q0 and Q1 (First Technology Safety Systems, Delft, The Netherlands), representing a 6-week-old and 1-year-old, respectively. Both were instrumented with custom sensor systems described below. In both experiments, participants were instructed to shake the relevant instrumented test dummy back and forth in its sagittal plane as violently as possible, and to keep that up for as long as they could, but with the emphasis on shaking as hard as possible. At the start and end of each shaking trial, the dummy was placed in a standardized posture in a frame, referred to as the homing bracket (see figure 1). Each participant shook a dummy twice, once sitting down with the dummy placed on their knee, and once standing up with the dummy held in front of them (see figure 1). Cases have been documented in which perpetrators have confessed to shaking an infant in approximately these positions [34,35].

The university’s Human Research Ethics Committee granted approval for both the Q0 and Q1 experiments (study numbers 3908 and 3408, respectively). All participants were adults and signed informed consent forms before partaking in the study. This research was performed in accordance with relevant guidelines and regulations. The participants shown in the figures in this article provided optional additional consent for use of recognizable footage in open access publications. Otherwise, all data were anonymized. The Q0 and Q1 experiments included 33 and 40 participants, respectively. The experiment using Q1 was completed first, and the Q0 experiment took place three months later. Participants were recruited separately for the two experiments. As a result, some participants partook in both experiments, but most only in one of the two.

### 2.2. Instrumented infant dummies

#### 2.2.1. Q0: 6-week-old infant

The Q0 dummy used in the current study is the same as was used by Schiks *et al.* in their study on shaking kinematics [29]. A custom sensor bracket was previously built to replace the Q0’s original sensor bracket, as described by Schiks *et al.* [29]. The instrumentation used there was adapted for the current experiment and consisted of one single-axis gyroscope (ADXRS649, measurement range ±20 000° s^−1^, Analogue Devices, Inc., Norwood Massachusetts, USA) in the head and torso each, oriented to record angular velocities in the sagittal *x–z* plane, and one tri-axial accelerometer (ADXL377, measurement range ±200 g, Analogue Devices, Inc., Norwood Massachusetts, USA) each at the head CoM and in the back of the torso (see figure 1). Both gyroscopes were modified to increase the maximum frequency response bandwidth of the sensor from 10 to 884 Hz to be able to capture high angular acceleration peaks, as described in supplementary materials A. The Q0 dummy had a total mass of 3.5 kg and a height of 485 mm. The mass of its head and neck together was 1.1 kg and the length of its neck was 55 mm.

#### 2.2.2. Q1: 1-year-old infant

Two identical sensor brackets were custom made to be mounted in the sensor spaces in the Q1 dummy; one in the head and one in the torso (photographs included in supplementary materials A). Each bracket contained one tri-axial accelerometer (ADXL377, measurement range ±200 g, Analogue Devices, Inc., Norwood Massachusetts, USA) and three gyroscopes (ADXRS649, measurement range ±20 000° s^−1^, Analogue Devices, Inc., Norwood Massachusetts, USA). Two of the gyroscopes were oriented to record angular velocities in the sagittal plane (rotation around the local *y*-axis), and one in the frontal plane (rotation around the local *x*-axis) (see figure 1). All gyroscopes were modified in the same way as in the Q0 dummy. The Q1 dummy had a total mass of 9.6 kg and a height of 740 mm. The mass of its head and neck together was 2.4 kg and the length of its neck was 74 mm.

### 2.3. Motion capture system

The experiments were conducted using an Oqus 700 motion capture system (Qualysis, Göteborg, Sweden). It consists of 12 infrared cameras that tracked the positions of spherical reflective markers fixed to the dummy. Clusters of four spherical ø16 mm infrared reflective markers were mounted to the head and torso of each dummy, using passive Traqr rigid-body clusters (Qualysis, Göteborg, Sweden). The reflective markers are indicated in figure 1. Motion capture data were recorded at the system’s maximum framerate of 500 Hz.

### 2.4. Data acquisition and processing

For both dummies, sensor outputs were recorded at a sample frequency of 5000 Hz using a data acquisition device (DAQ) (NI USB-6211, National Instruments, Austin, TX, USA) connected to a laptop. All sensor data and the optical motion capture data were synchronized using a trigger signal provided by the Qualisys motion capture system. All data were processed and analysed using MATLAB (Version R2023b, MathWorks, Natick, MA, USA).

### 2.5. Data analysis

#### 2.5.1. Sensor data

The variables obtained from the sensor data were the following:

— *ω*_h_*, ω*_t_ (rad s^−1^): Angular velocity of the head and torso in their local sagittal planes. Obtained from sagittal gyroscopes in head and torso, respectively.
— *α*_h_*, α*_t_ (rad s^−2^): Angular acceleration of the head and torso in their local sagittal plane. Computed through differentiation of *ω*_h_ and *ω*_t_ over time, respectively.
— *a_xh_, a_xt_, a_zh_, a_zt_* (m s^−2^): Linear acceleration of the head and torso in the *x*-direction (forward) and the *z*-direction (superior), respectively. Obtained from a three-axis accelerometer in the head and torso.

The equations of motion are included in supplementary materials B.

#### 2.5.2. Motion capture data

Motion capture data consisted of three-dimensional coordinates over time of four torso markers and four head markers. The head marker cluster and torso marker cluster were treated as rigid bodies. Marker occlusions lasting fewer than 10 frames were interpolated using the Qualisys Track Manager (Qualysis, Göteborg, Sweden) default polynomial interpolation. When marker occlusions longer than 10 frames occurred, missing marker coordinates were computed based on the position of the remaining three markers. No marker occlusions of more than one marker per cluster and lasting longer than 10 frames occurred during the shaking trials.

#### 2.5.3. Local head and torso reference frames

The locations of origins of the head and torso reference frames, where the accelerometers are located in the head and torso (see figure 1*e*–*g*), could not be directly recorded using the optical motion capture system during shaking, as markers inside the dummy would be occluded. The accelerometer positions were therefore added as virtual markers during post-processing. To this end, the orientation and offset of the marker cluster with respect to the accelerometer location was determined during a stationary measurement in which the accelerometer locations and orientations could be indicated using markers. These were then used to determine the orientation of the local reference frame and to add the virtual markers to each frame of the shaking motion capture data. The steps are described in detail in supplementary materials C.

#### 2.5.4. Projection to primary shaking plane

**Figure 2.**
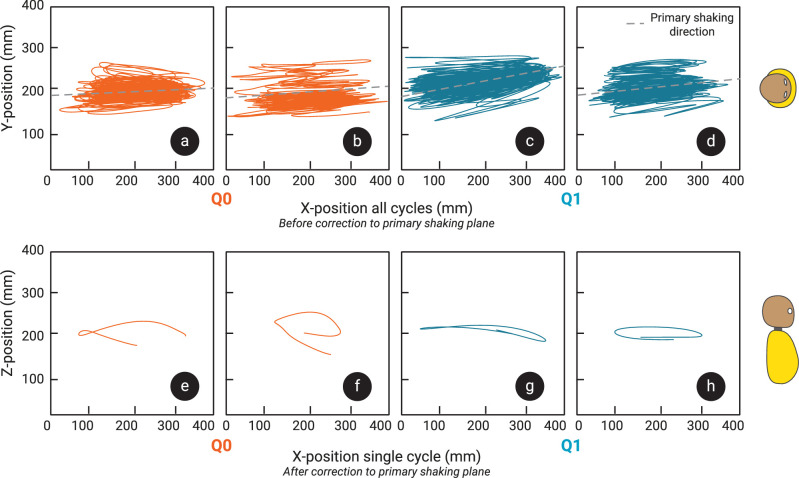
Typical examples of trajectories of the head’s local reference frames origin in Q0 (a,b) and Q1 (c,d) in standing up (a,c) and sitting down (b,d) posture in the global *x–y* plane (top view of the dummy). This shows that shaking occurs mostly in one direction, indicated as the primary shaking direction by the dashed line. Typical examples of the head origin trajectories of a single shaking cycle in the primary shaking plane over time from the Q0 dummy (e,f) and Q1 dummy (g,h), in standing up (e,g) and sitting down (f,h) postures show varied trajectory shapes. Corresponding views of the dummy are shown on the right-hand side of the figures to illustrate the corresponding top view (a–d) and sagittal view (e–h) of the dummy.

Accelerations in the lateral direction of both head and torso (local *y*-axes) were found to have negligible magnitude compared to those in the sagittal planes (spanned by local *x*- and *z*-axes). Therefore, it was decided to analyse the motion capture data by projecting to a single plane. A ‘primary shaking plane’ was defined for each trial by determining the plane spanned by the gravity vector and the mean direction of the velocity of the torso in the global reference frame (see examples in figure 2). The global reference frame was then rotated such that the primary shaking plane aligned with the *x*–*z* plane of the global reference frame. Motion trajectories were then projected on to this primary shaking plane for further analysis.

#### 2.5.5. Shaking cycles

The data from each trial were sliced into individual shaking cycles, defined as one complete back and forth motion of the dummy. Data were sliced at the middle between maxima and minima of the torso’s *x*-position (forward in shaking plane) in the global reference frame, so that each peak is only captured in one cycle. For each trial, the cycle with the highest peak angular acceleration of the head in its sagittal plane was selected. We refer to this cycle as the *fiercest cycle*, though this is, of course, an arbitrary definition of ‘fiercest’ and does not necessarily imply ‘most harmful’.

#### 2.5.6. Shaking characteristics

The mean shaking frequency was computed by averaging the number of shaking cycles per second over the whole trial. The shaking amplitude of the torso was computed for each cycle as the distance in the primary shaking plane between the torso’s origin at its maximum and minimum *x*-position in the shaking plane within that cycle. This was repeated for the head to compute the shaking amplitude of the head for each cycle. Peak neck flexion (ventral, positive) and extension (dorsal, negative) were determined during each cycle by computing the angle between the *z*-axes of the head and torso, in the torso’s local sagittal plane, with respect to the dummy’s neutral pose as shown in figure 1*e*–*g*. The mean peak neck flexion and extension were computed for each trial. All these shaking characteristics were based on data from the optical motion capture system.

#### 2.5.7. ICOR

The ICOR of the head in the primary shaking plane was computed for all samples in the fiercest shaking cycle using the following steps for each frame, based only on the optical motion capture data:

(1) Compute normal vectors to the curve of the trajectories of two points on the head (point 1: head’s local reference frame origin and point 2: mean position of head’s marker cluster)
(2) If the angle between the normals at these two points is less than 5° (threshold selected somewhat arbitrarily, to be sure lines are not collinear, use another point on the head (point 2 replaced by location of head’s vertex), as this small angle may have two causes:
  (a) Either the selected points on the head are close to collinear with the ICOR, making the ICOR calculation inaccurate owing to high sensitivity to noise and inaccuracies. This will be solved by using a different point on the head, as this point can never *also* be collinear with the ICOR.
  (b) Or the ICOR is very far from the head, which will still be detected using a different point on the head.
(3) Compute the intersection point of the two normals. This point is defined as the ICOR [36]. We define the head IROR as the distance between the ICOR and the head’s local reference frame origin. Note that the estimated ICOR is less accurate for large values of the IROR; for a large IROR, small inaccuracies in the slopes of the trajectories will have a large effect on the estimated ICOR position. However, this is not critical, since head motion is predominantly translational at large IRORs.

For a graphical illustration of these steps, refer to fig. 4 in Schiks *et al.* [29].

### 2.6. Data filtering

The sensor data contained low-power high-frequency noise. Therefore, the accelerometer data were filtered using a twelfth-order low-pass zero-phase-lag digital IIR Butterworth filter with a frequency bandwidth of 500 Hz. A power spectral density analysis showed peaks at 50 Hz and its harmonics, which were probably the result of signal pollution from the main electric power through the power supply used for the sensors. The 50 Hz peak was filtered out using a second-order digital IIR Butterworth notch filter with a 3 dB bandwidth of 50 Hz, and its harmonics were each filtered out using the same notch filter except with a 3 dB bandwidth of 2.5 Hz. The same twelfth-order low-pass Butterworth filter at 500 Hz was applied to the angular velocity output of the gyroscope.

The optical motion capture trajectories needed to be smoothed, since spikes and jitters in these trajectories would make it impossible to fit tangent lines to compute the ICOR. A power spectral density analysis revealed that the power beyond 10 Hz was less than −25 dB, and the majority of the signal was between 3 and 5 Hz. Therefore, motion capture data was filtered using the same twelfth-order Butterworth filter, except using a cutoff frequency of 30 Hz to provide margin.

### 2.7. Statistics

Statistical analyses were conducted using IBM SPSS Statistics 26 to check for effects of experimental conditions and participant characteristics on the following outcome measures per trial:

— Total shaking duration (s)
— Mean shaking frequency (Hz)
— Mean peak neck-flexion (°)
— Median IROR of head with respect to its origin during fiercest shaking cycle (mm)
— For head and torso each:
  — Mean amplitude at accelerometer location (mm)
  — Mean peak resultant linear acceleration in the sagittal plane at the centre of the accelerometer (m s^−2^)
  — Mean peak angular acceleration in the sagittal plane (rad s^−2^)

The following tests were conducted:

— Mann–Whitney U tests for significant difference in outcome measures between trials with Q0 or Q1 dummy, and between trials with a female or male participant
— Wilcoxon signed-rank test for significant difference in outcome measures between paired trials in standing up or sitting down posture
— Spearman correlation for significant correlations between outcome measures and participant age or participant height

All analysed data were examined for normality and Anderson–Darlings tests indicated almost all were not normally distributed (see supplementary materials E), which is why non-parametric tests were applied. Bonferroni correction was used to adjust the significance level for multiple testing (*α* = 0.005).

### 2.8. Checking neck failure effect

The neck of the Q0 dummy failed after ‘use’ by 35 participants. The neck consisted of a stack of four steel discs with flexible moulded rubber between the discs and a tensioned multicore steel cable running longitudinally through its centre. The steel cable was found to be severed, allowing the rubber between the two proximal discs to tear, separating head from trunk. Since the neck may have gradually degraded over the course of the trials, this could have affected the neck stiffness. We therefore wished to investigate whether there was any significant change over the course of the trials in the head amplitude, head peak resultant linear acceleration and angular acceleration, or the median IROR. Spearman’s correlations were computed between each of these outcome measures described above and the chronological number of the trial to find if any of the outcome measures significantly increased or decreased over the course of the experiment.

## 3. Results

### 3.1. Overview

Motion capture, accelerometer and gyroscope data were acquired for the head and torso of the test dummies. Q0 was shaken by 33 participants (18 female, 15 male) and Q1 by 40 participants (18 female, 22 male), with respective mean ages of 34 (range 23–69) and 32 (range 21–63) years old and mean heights of 176 cm (range 158–196 cm) and 176 cm (range 162–197 cm). Two of the original 35 participants in the Q0 set were excluded, one owing to a failure in the data-recording set-up resulting in missing data, and one owing to failure of the dummy neck. No further trials were recorded after failure of the neck. In general, participant characteristics were found to have a limited effect on the shaking kinematics, while posture (standing up versus sitting down) and the dummy itself (Q0 versus Q1) had a more apparent effect. All outcome measures, combined and split by dummy, participent sex, shaking and posture are included in supplementary materials D, and outcomes of statistical tests are included in supplementary materials E.

### 3.2. Neck failure

Since the neck may have gradually degraded over the course of the trials before actually breaking, this could have affected performance, making it less stiff over time. If so, this would probably result in higher peak accelerations of the head. However, no significant systematic increase or decrease was found over the course of the trials in head amplitude, shaking frequency, head peak resultant linear acceleration and rotation acceleration, or the median IROR (see supplementary materials E). Therefore, we can assume that the failure of the neck was instantaneous and hence did not affect the outcomes of this study.

### 3.3. Motion pattern

**Figure 3.**
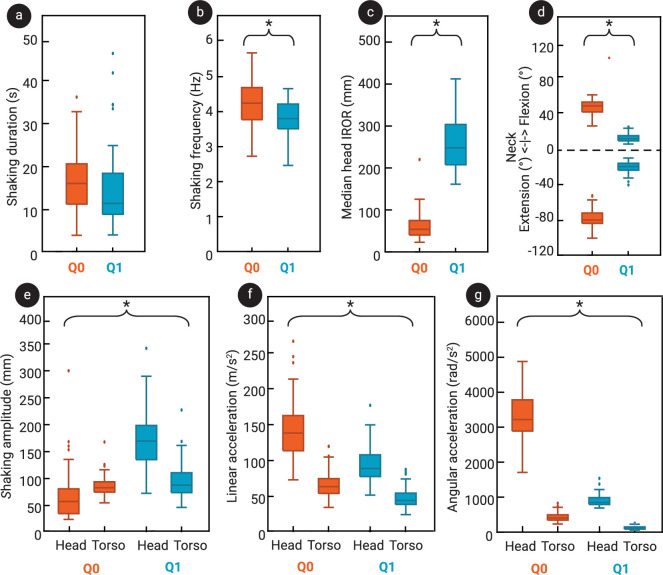
Distributions of the shaking duration (a) and shaking frequency (b) across all trials, shown split by dummy. Distributions are also shown of the median head IROR (c) for the fiercest cycles of all trials, split by dummy. (d) The distribution of peak neck flexion and extension across all trials, split by dummy. Shaking amplitude (e), linear acceleration (f) and angular acceleration (g) are shown for both head and torso, split by dummy. Stars indicate significant differences were found between Q0 and Q1 distributions.

Some significant variation was found in the shape, magnitude and direction of head origin trajectories in the primary shaking plane, both across and within trials. Typical examples of head trajectories from isolated shake cycles in the primary shaking plane are presented in figure 2*e*–*h*. Note that the cycles do not necessarily start and end in the same position, as each cycle is slightly different and cycle slicing is based on torso *x*-position. Statistically significant differences were found between Q0 and Q1 in head amplitude and shaking frequency (*p* < 0.001 and *p* < 0.001). The median head shaking amplitude in Q0 was 56.6 mm (s.d. 47 mm), while in Q1, it was 171.8 mm (s.d. 51 mm). Mean peak neck flexion/extension also differed significantly between the dummies, with Q0 reaching much larger deflections at a median of 79.5° (s.d. 15°), than Q1, which showed 18.8° (s.d. 7°). No significant differences in torso amplitude or shaking duration (*p* = 0.091 and *p* = 0.009) were found between the dummies. Shaking amplitudes, frequency, duration and neck flexion/extension, corresponding to each dummy, are given in figure 3. Paired testing showed no significant difference in head or torso amplitudes, frequency, duration or neck flexion between the standing up and sitting down trials. The median shaking duration across all trials was 13.3 s (s.d. 8 s).

No significant differences were found between men and women in amplitudes, frequency, duration or neck flexion, and no effect of participant age was found on any of these. Height was found to have a weak negative significant correlation (−0.243) with torso amplitude (*p* = 0.003). Head amplitude, frequency, duration or neck flexion was not significantly correlated with participant age.

### 3.4. Accelerations

**Figure 4.**
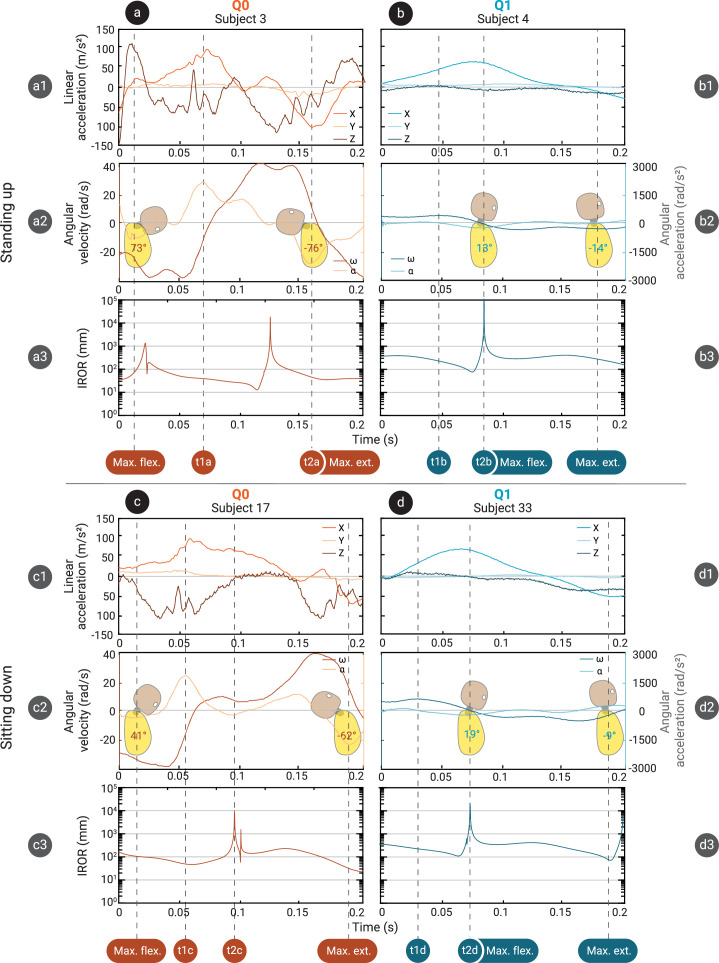
Four typical examples of data from a single fiercest shaking cycle for Q0 (a,c) and Q1 (b,d) from standing up (a,b) and sitting down (c,d) trials. Head linear accelerations (a1–d1), head angular acceleration α and velocity ω (a2–d2) and IROR with respect to the head’s local reference frame origin (a3–d3) are shown for each trial. Some timestamps of interest are also marked for each trial by vertical dashed lines. t1a, t2a: Peak angular accelerations correspond to low sections of the IROR. t1b: peak angular acceleration corresponds to low section of IROR. t2b: peak IROR corresponds to high linear acceleration and low angular velocity and acceleration. t1c: Peak angular acceleration corresponds to low IROR. t2c: peak IROR corresponds to fairly high linear acceleration and low angular velocity and acceleration. t1d: Peak angular acceleration corresponds to a fairly low region of IROR. t2d: Peak IROR corresponds to high linear and low angular acceleration. Icons of the dummy are included showing neck flexion angles at timestamps of maximum and minimum flexion.

**Figure 5.**
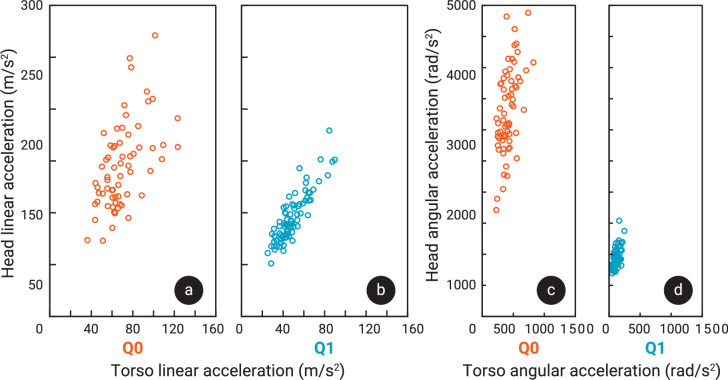
Scatter plots of the head’s peak resultant linear acceleration in the sagittal plane per trial against the corresponding torso peak linear acceleration, for Q0 (a) and Q1 (b). The same scatter is shown for the peak angular accelerations in the sagittal plane for Q0 (c) and Q1 (d).

Typical examples of the linear accelerations and angular accelerations and velocities in the sagittal plane of the Q0 and Q1 head during a single shaking trial are shown in figure 4. In general, higher torso accelerations resulted in higher head accelerations for both dummies, with a tighter trend for Q1 and higher accelerations with more variability for Q0 (see figure 5). Mean peak angular and resultant linear acceleration in the sagittal plane of both head (*p* < 0.001) and torso (*p* < 0.001) were significantly higher in Q0 than in Q1 (figure 3). Resultant sagittal linear accelerations of head and torso in Q0 were 140.2 m s^−2^ (s.d. 41 m s^−2^) and 63.5 m s^−2^ (s.d. 19 m s^−2^), respectively, while in Q1 these were, respectively, 89.3 m s^−2^ (s.d. 24 m s^−2^) and 43.7 m s^−2^ (s.d. 14 m s^−2^). The mean *z*-accelerations of the head across trials were −19.7 m s^−2^ (s.d. 13 m s^−2^) in Q0 and −17.6 m s^−2^ (s.d. 6 m s^−2^) in Q1. The sign of the *z*-accelerations was negative 70.5% (s.d. 13%) of the time in Q0 and 94.2% (s.d. 6%) in Q1. Examples of shaking cycles in which the *z*-accelerations were almost continuously negative or more varied are shown in figure 4*a*–*d*. The difference in angular accelerations was more striking, with angular accelerations in Q0 reaching 3218.4 rad s^−2^ (s.d. 683 rad s^−2^) in the head and 408.2 rad s^−2^ (s.d. 126 rad s^−2^) in the torso, while in Q1 these were 846.6 rad s^−2^ (s.d. 159 rad s^−2^) and 104.9 rad s^−2^ (s.d. 45 rad s^−2^), respectively. In paired comparison, no significant difference between sitting down and standing up trials was found for linear or angular rotation of the head or torso.

No significant correlations were found between participant age and any of the acceleration outcome measures. Significant correlations with participant height were found for the mean peak linear resultant sagittal accelerations of the head and torso (*p* < 0.001 and *p* < 0.001), though both correlations were weak (coefficients of 0.29, 0.29). Although participants’ weights were not recorded, we tentatively observed that participants with a higher body mass tended to be able to shake more fiercely, particularly with Q1.

### 3.5. Instantaneous centre of rotation

**Figure 6.**
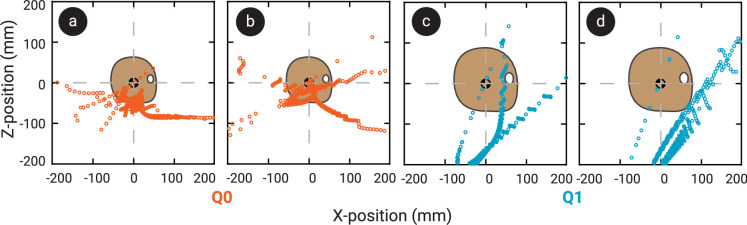
Typical examples of all the locations of the ICOR over the course of a single fiercest shaking cycle for Q0 (a,b) and Q1 (c,d) in standing up (a,c) and sitting down (b,d) postures, expressed in the head’s local reference frame.

**Figure 7.**
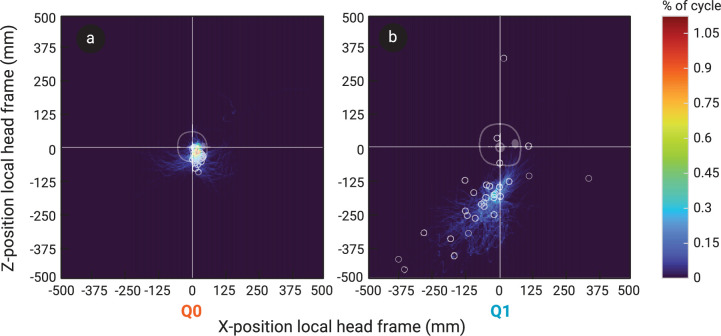
Heatmaps indicating for each *x*–*z* position which % of time during each fiercest cycle (% of cycle) the ICOR was at that location. This is shown only for each fiercest cycle from all 66 trials in Q0 (a) and 80 trials in Q1 (b), expressed in the head’s local reference frame. The overlain scatter plot with light grey circles shows the locations of the ICOR at the moment of the peak angular acceleration for each fiercest cycle. The silhouettes of the dummy heads are to scale with respect to the axis scales.

The ICOR of the head in the primary shaking plane moved through space over time. Some typical examples of the locations of the ICOR with respect to the dummies’ heads during a single shaking cycle for both Q0 and Q1 are given in figure 6. The IROR consequently also varied a lot over the course of a single shaking trial for both dummies, from close to zero (almost pure rotation) to 10^4^ m (almost pure translation). Peaks in linear or angular acceleration did not necessarily coincide with local maximum or minimum values of the IROR. A typical example of the magnitude of the ICOR over time during a single shake cycle is given in figure 4. The median IROR during the fiercest shaking cycle was significantly smaller in Q0 than in Q1, with a radius of 53.8 mm (s.d. 31 mm) in Q0 and 247.9 mm (s.d. 56 mm) in Q1 (see figure 3). This means that the ICOR tended to be closer to the centre of the head during shaking in Q0, whereas in Q1, it tended to be farther away. This is also illustrated in the heatmap in figure 7, which shows the ICOR locations across all fiercest cycles in Q0 and Q1, as well as the locations of the ICOR at peak head angular accelerations. Figure 7 shows that the ICOR at peak head angular sagittal acceleration tended to be closer to the centre of the head in Q0 than in Q1, across all trials. Note that the plot range is set to a somewhat arbitrary range of ±500 mm, as the position of the ICOR is most interesting at smaller distances from the head CoM, and this is where the ICOR was located the majority of the time. Participant height and age were not found to correlate significantly with the median IROR in the fiercest shaking cycles, and no significant difference in IROR was found based on participant sex.

## 4. Discussion

The goal of the current study was to record the kinematics of participants shaking two infant dummies, representing a 6-week-old (Q0) and a 1-year-old (Q1) infant, to identify differences in shaking kinematics based on the characteristics of both the dummies and the person shaking. As expected, participants were able to induce higher accelerations when shaking the smaller Q0 than the larger Q1 dummy. Linear accelerations of the head and torso were both higher in Q0 than in Q1, and this difference further widened for the angular accelerations (figure 3*f*,*g*). The median IROR during Q0 shaking was lower than in Q1, meaning the centre of rotation tended to be closer to the head in Q0 during the shaking cycles that included the highest peak head angular accelerations than in Q1 (figure 3*c*). Moreover, the peak angular accelerations of the Q0 head were accompanied by ICORs closer to the head’s origin than in Q1 (figure 7). In short, the motion in Q0 was more rotational in nature than in Q1.

Several studies have investigated the kinematics of an infant model’s head during shaking. Schiks *et al.* used almost the same instrumentation as in the current study to record the kinematics of shaking a Q0 dummy and found mean peak linear head CoM accelerations of 149 m s^−2^ (s.d. 48 m s^−2^) [29]. Stray-Pedersen *et al*. similarly recorded shaking kinematics using a Q0 dummy and found mean peak linear accelerations at the skull centre of 182 m s^−2^ (range 64–335 m s^−2^) in the anterior–posterior direction (defined as *x*-direction in the current study) and −172 m s^−2^ (range −51 to −431 m s^−2^) in the superior−inferior direction (defined as *z*-direction in the current study) [37]. In the current study, we also found that *z*-accelerations of the head were negative the majority of the time, for both Q0 and Q1. Nadarasa *et al.* also recorded kinematics of a Q0 during shaking and found linear resultant accelerations in the range of 93–120 m s^−2^ [38]. Note that studies on shaking kinematics often report linear accelerations at the vertex or occiput, rather than at the CoM as we did here, and that accelerations at the vertex and occiput are generally much higher because the ICOR is generally below the head CoM. For example, Glowinski *et al.* recorded a dummy’s linear head kinematics during shaking and found peak linear occiput accelerations of 410 m s^−2^, while peak linear accelerations at the neck were 154 m s^−2^ [39]. In the current study, we found a median peak linear head acceleration of the Q0’s head CoM of 140 m s^−2^ (s.d. 42 m s^−2^). In short, the measurements in our study and those found in the literature are in close agreement, and recent literature generally shows strong agreement in the order of magnitude of peak linear head accelerations of small infant dummies during violent shaking.

The neck of the Q0 dummy failed after recording 35 participants. If the neck degraded over the course of the trials and not all at once, this would have affected the neck stiffness, with reducing stiffness over progressing trials. However, since no systematic increase or decrease in outcome measures was found over the course of the experiment, it is assumed that the neck failure did not influence our conclusions. In addition, the biofidelity of the Q0 and Q1 dummies is not entirely known, with the accuracy of the neck stiffness in particular often being questioned. These dummies were designed for high-speed applications such as car crashes, and the necks therefore appear to be too stiff when subjected to the lower accelerations involved in fierce shaking. However, since the goal of the current study was to compare two dummies, and not to obtain absolute estimates of head accelerations that would occur in a real infant, this uncertain biofidelity does not affect the outcomes of the study. Moreover, the neck stiffness of actual infants is probably lower than in the dummies, particularly for Q0. Since it has been shown that a more flexible surrogate neck leads to higher angular head accelerations [40], the head accelerations recorded in the current study would probably be even higher in actual infants subjected to the same shaking input. One explanation for the differences in the head’s kinematics between Q0 and Q1 is that the larger size and mass of Q1 makes fierce shaking more physically exerting, and for a weaker person, potentially even impossible. We found that the dummy motions applied to the torsos of the dummies resulted in higher angular accelerations of the torso in Q0 than in Q1 (figure 5*c*,*d*), probably because its inertia made it much easier to induce a rotational motion from the wrist. Second, owing to Q0’s smaller dimensions, and particularly owing to its shorter neck, the same input motion on the torso would result in a smaller IROR for the head in Q0 than in Q1, further emphasizing the increased risk in younger/smaller infants. Finally, Q1 has a stiffer neck than Q0, partly explaining the much lower peak neck flexion angles (figure 3*d*), and it has been shown that head accelerations in shaking are lower for stiffer necks with the same torso input [16,19]. The higher torso angular accelerations in Q0 would have induced higher head angular accelerations even without the difference in neck stiffness, though the weaker neck of Q0 probably exacerbated the difference.

The observed difference in shaking kinematics between Q0 and Q1 may align well with the fact that IHI-ST incidence and mortality are highest in the first months of life [1,6–10]. There have been various thoughts on why, including that an older infant may be less likely to be shaken at all, since infants cry most during the first 3 months of life, and crying is often brought up as a reason for shaking in confessed cases [41]. Second, tissues of older infants may have higher injury tolerances, so their more mature tissue may be less likely to incur injury under the same stresses. Results from the current study suggest that, on top of those reasons, the kinematics of shaking a younger infant result in more rotational motion of the head (figure 3*c*,*f*,*g*), which has been thought to be an important mechanism responsible for injuries associated with IHI-ST [11,30–32]. Using their finite element model of an eye, Song *et al.* found higher traction stresses occurred at the vitreoretinal interface during rotational motion than during linear motion [28]. Complex retinal haemorrhages are characteristic of IHI-ST and typically consist of splitting of the delicate layers of the retina (retinoschisis) and scattered haemorrhages around the whole fundus [42]. It may be hypothesized that rotational motion of the brain with respect to the skull, e.g. resulting from a motion with a short IROR, could put increased pressure or tension on the optic nerve, thereby potentially exacerbating the tension stresses in the retinal layers [28,42]. Moreover, rotation of the brain relative to the skull has been hypothesized to induce damaging shear in, for example, the bridging veins, and to result in higher local strain within the brain tissue [11,30–32]. This implies that having the ICOR closer to the centre of the head in Q0 compared to Q1 suggests there will be more rotation of the brain with respect to the skull, and hence a higher potential of injury to bridging veins and brain matter in younger infants.

Although participants’ weights were not recorded, we observed that participants with a higher body mass tended to be able to shake more fiercely, particularly with Q1. This may be explained by the imbalance in inertia between lighter participants and the relatively heavy Q1 dummy, making it more strenuous to accelerate the dummy back and forth without moving themselves back and forth equally. For application in actual cases in suspected shaking, it would be interesting if a relation could be found between perpetrator physique and fierceness of shaking. In future work, it may be insightful to further characterize participants by also recording weight and level of fitness and to include a wider range of participant ages, physiques and backgrounds. Moreover, it may be useful to query participants on, for example, whether they shook as hard as they could, and how physically strenuous they found it. In addition, the age/weight represented characteristics of the dummy clearly affected the kinematics of shaking it. Application of a dummy for reconstruction and measurement of shaking and other traumatic events in forensic investigations as well as in research should therefore preferably be done using a dummy that matches the victim’s physique as closely as possible. One way of achieving such functionality would be by developing an instrumented dummy with adjustable physique and joint properties. Such an approach is ongoing work by the authors.

To the best of our knowledge, no study has previously recorded and reported the complete kinematics of both the head and torso of an infant dummy during shaking using a moving ICOR model. Schiks *et al.* [29] have shown that using a fixed ICOR model results in significant inaccuracies in estimated head angular accelerations based on recorded linear accelerations. The kinematics reported in the current study may therefore be useful as input in performing biomechanical modelling studies of IHI-ST in the future, as not doing so may result in the underestimation of potentially harmful kinematics of shaking an infant, particularly those resulting from angular accelerations.

To conclude, the current study showed that:

— As expected, participants induced higher linear and particularly angular accelerations on both head and torso of Q0 during shaking than in Q1, or simply put: participants could shake Q0 more violently than Q1.
— Higher peak angular accelerations of the Q0 head during shaking were accompanied by smaller IRORs with respect to the head origin, suggesting a higher potential for injurious shear of intracranial tissues than in Q1.
— Because it has been suggested in the literature that sagittal angular acceleration of the head is an important mechanism in inducing the injuries associated with IHI-ST [11,30–32], the results of this study show that shaking a smaller/younger infant is more likely to cause the kinematics possibly responsible for IHI-ST.

## Data Availability

The dataset generated during this project (except recognisable video footage of participants) has been made available as an open access dataset in the 4TU Centre for Research Data Repository [43]. For questions about the dataset and access, contact the corresponding author. Supplementary material is available online with this publication [44].
